# Synthesis, Characterization, and Properties of a Novel Hyperbranched Polymers with Polyacrylamide Side Chains

**DOI:** 10.3390/ma17071619

**Published:** 2024-04-01

**Authors:** Xiaoping Qin, Qianwen Wang, Peng Tang, Hui Yang, Cuixia Li, Xiaoliang Yang, Tong Peng

**Affiliations:** 1 School of Chemical Engineering, Sichuan University of Science and Engineering, Zigong 643000, China; 13568322767@163.com (X.Q.); 18982607289@163.com (Q.W.); licuixia2023@126.com (C.L.); 2 Tianjin Key Laboratory of Brine Chemical Engineering and Resource Eco-Utilization, College of Chemical Engineering and Materials Science, Tianjin University of Science and Technology, No.29, 13th Street, Binhai New District, Tianjin 300457, China; 3 Jidong Oilfield Branch Company, PetroChina Company Limited, Tangshan 063002, China; yanghui2023jd@outlook.com (H.Y.); yangmeimei178@163.com (X.Y.); jd_pengtong@petrochina.com.cn (T.P.)

**Keywords:** hyperbranched polymers, amphiphilic polyacrylamide, polymerization, thickening ability, enhanced oil recovery

## Abstract

A novel hyperbranched polymer with polyacrylamide side chains (HAPAM) was synthesized by aqueous solution polymerization using acrylic acid, acrylamide, 2-acrylamido-2-methyl-1-propanesulfonic acid, hydrophobic monomer of dimethyl octadecyl ammonium chloride, and the homemade skeleton monomer of modified-M_2.0_ as raw materials and (NH_4_)_2_S_2_O_8_-NaHSO_3_ as initiator. The molecular structure, functional groups, and surface morphology of HAPAM were characterized by Fourier transform infrared spectroscopy, nuclear magnetic resonance hydrogen spectroscopy, and scanning electron microscopy. It was found that the performance of HAPAM solution was higher than that of ordinary polyacrylamide solution in terms of thickening ability, shearing resistance, thermal endurance, salt-resistance, resistance-coefficient and residual-resistance-coefficient, ability to reduce interfacial tension between polymer solution and crude oil, and oil-displacement-efficiency. In particular, the enhanced oil recovery of the HAPAM solution was 13.03%, and the improvement of shearing resistance and immunity to chromatographic separation were simultaneously achieved by the HAPAM solution. These results indicate that the successful synthesis of the novel HAPAM opens a promising strategy for developing new high-performance oil-displacing polymers.

## 1. Introduction

After the first and second stages of oil production from the well, there were still large volumes of the original oil bypassed or trapped in the reservoir [1,2,3]. Therefore, enhanced oil recovery (EOR) methods were used to recover the additional oil [4,5,6,7]. The EOR methods were majorly classified into thermal and non-thermal EOR [8,9,10]. Unfortunately, thermal EOR methods were unsuitable for reservoirs with great depth and thin pay zones [11]. In addition, the application of thermal EOR was still challenged both economically and environmentally because of the high cost of heat supply along with excessive CO_2_ emission and costly post-treatment and maintenance [12,13]. Hence, non-thermal EOR methods, including physical, chemical, and biological techniques [14], have received prodigious attention in the recovery of conventional and heavy oil [15]. Amongst all non-thermal EOR methods, chemical EOR has been recognized as the most promising technique due to its technical and economic feasibility and reasonable capital cost [16]. Polymer flooding was the simplest and most widely used chemical EOR process [17].

Because of the synergistic improvement of sweep efficiency and oil-washing efficiency, binary polymeric surfactant mixtures (BPSMs) have become the main chemical EOR technique for recovering the oil in oilfields with high and ultra-high water cuts [18,19]. However, the on-site applications were still severely inhibited due to two major technical bottlenecks. First, the extensively used BPSMs (such as partially hydrolyzed linear polyacrylamide, PAM) still have relatively poor shear resistance [20], and their sweeping efficiency and oil-washing efficiency were low [21], which need to be further advanced. Second, the synergistic effect of BPSMs was significantly weakened by chromatographic separation (i.e., the differential transport speed of BPSMs flowing in the boreholes after injection into the stratum). Therefore, it is necessary to improve the poor shear resistance and avoid the chromatographic separation of BPSMs to further advance their oil-displacing performance [22,23].

In recent years, several research groups reported that oil-displacing polymers with special molecular structures (comb-type [24], star-type [25], and hyperbranched type [26]) show strong shear resistance [27,28]. Duan et al. [29] studied the flow-induced scission behavior of PAM with four-arm-star during planar elongational flow in a cross-slot flow cell. They also found that the PAM with a four-arm star exhibited better-shearing stability than that of the linear PAM at the same shearing rate. Chen et al. [30] synthesized a new type of chitosan-modified hyperbranched polymers (HPDACs) by the free-radical polymerization of surface-modified chitosan with acrylic acid (AA) and acrylamide (AM). After mechanical shearing, the viscosity retention rate of HPDACs in ultrapure water and simulated brine was higher than that of PAM and the dendritic polymer, indicating the excellent shear resistance and good viscoelasticity of HPDACs. In addition, in order to effectively avoid chromatographic separation problems of BPSMs, both hydrophobic and hydrophilic groups were introduced into BPSMs [31] to improve the ability to expand the swept volume and oil displacing efficiency [32,33]. Zhu et al. [34] synthesized an amphiphilic polymer with twin-tailed hydrophobic groups (TAP). They estimated a lower critical aggregation concentration (CAC, 800 mg/L) of TAP, and a higher apparent viscosity can be obtained when the concentration of TAP was higher than CAC due to the strong hydrophobic association. However, to date, there have been limited reports about hyperbranched amphiphilic polyacrylamides (HAPAMs) with synergistic effects of excellent shearing resistance and a strong ability to reduce interfacial tension [35,36,37,38,39,40]. To this end, in this work, a novel HAPAM with 16 branched chains was successfully synthesized by introducing hydrophobic group (dimethyl octadecyl ammonium chloride, DMCAAC-18) and hydrophilic groups (AA, AM, and AMPS, abbreviated from 2-acrylamido-2-methyl-1-propanesulfonic acid) into the molecular chain, simultaneously enhancing the shear-resistance and avoiding the phenomenon of chromatographic separation. Their microstructures, functional groups, and surface morphology were characterized using Fourier transform infrared spectroscopy (FT-IR), nuclear magnetic resonance hydrogen spectroscopy (1H-NMR), and scanning electron microscopy (SEM). The differences between HAPAM and PAM on thickening ability, shear-resistance, temperature resistance, salt resistance, resistance-coefficient and residual-resistance-coefficient, ability to reduce interfacial tension, and oil-displacement-efficiency were investigated via a series of systematic tests.

## 2. Experimental Section

### 2.1. Materials

All chemicals used in this study were of analytical grade and were used without any further purification. Acrylamide (AM), acrylic acid (AA), sodium hydroxide, sodium bicarbonate, ethanol, sodium chloride, potassium chloride, sodium bisulfite (SDS), ethylenediamine (EDA), methanol, methyl acrylate (MA), N,N-Dimethylformamide (DMF), Maleic anhydride (MAH) and anhydrous sodium sulfate were purchased from Chengdu Kelong Chemical Co., Ltd. (Chengdu, China). 2-acrylamide-2-methyl-1-propane sulfonic acid (AMPS) was purchased from Bide Pharmatech Co., Ltd. (Shanghai, China) Ammonium persulfate was supplied by Guangdong Guanghua Sci-Tech Co., Ltd. (Shantou, China). Anhydrous magnesium chloride was purchased from Tianjin Balance Bio-tech Co., Ltd. (Tianjin, China). Calcium chloride was supplied by Chengdu Jinshan Chemical Reagent Co., Ltd. (Chengdu, China). Dodecyl dimethyl allyl ammonium chloride (DMDAAC-18) was bought from Zhangjiagang Renda Chemical Co., Ltd. (Suzhou, China). And ammonium persulfate (APS) was supplied by Guangdong Guanghua Sci-Tech Co., Ltd. (Shantou, China). Especially, the composition of the simulated injection-water was given in Table 1.

### 2.2. Synthesis Processes

#### 2.2.1. Synthesis of Modified-M_2.0_

As described in the reference [41], the skeleton-polymer of a 2.0 generation dendritic macromolecule (M_2.0_) with eight branch chains was synthesized as follows steps. Step (i), 18.0 g of ethylenediamine (EDA) and 60 g of methanol were added into a three-necked flask with a magnetic stirrer for stirring, a reflux condenser for reflux condensation, and a thermometer for temperature-monitoring. In addition, 113.2 g of methyl acrylate (MA) was gradually dripped into the solution. The reaction was controlled at 25 ± 0.1 °C for 24 h. After the reaction, M_0.5_ was purified and obtained by vacuum distillation and silica gel column. Step (ii), 40.4 g of M_0.5_ and 64 g of methanol were added into a fresh three-necked flask with a magnetic stirrer, reflux condenser, and thermometer, dripping with 80 g of EDA. The reaction of the stirring solution was controlled at 72 ± 0.1 °C for 24 h. After the reaction, M_1.0_ was purified and obtained by vacuum distillation and silica gel column. Step (iii), 30 g of M_1.0_ and 40 g of methanol were added into a fresh three-necked flask with a magnetic stirrer, a reflux condenser tube, and a thermometer, with dripping 50 g of MA. The reaction of the stirring solution was controlled at 72 ± 0.1 °C for 24 h. After the reaction, M_1.5_ was purified and obtained by vacuum distillation and silica gel column. Step (iv), 30 g of M_1.5_ and 50 g of methanol to a fresh three-necked flask with a magnetic stirrer, reflux condenser tube, and a thermometer, dripping with 14 g of EDA. The reaction of the stirring solution was also controlled at 72 ± 0.1 °C for 24 h. Finally, the dendritic skeleton-polymer of M_2.0_ was purified and obtained by vacuum distillation and silica gel column. Step (v) was the process of modifying the structure of M_2.0_. 8 g of M_2.0_ and 30 g of N, N-Dimethylformamide (DMF) were added into a beaker to dissolve M_2.0_ completely, slowly dripping with 4.4 g of maleic anhydride. After finishing the addition of maleic anhydride (MAH), the beaker was sealed and put into an oven to react at 70 °C for 6 h. In the end, the solution of the modified-M_2.0_ was obtained in the form of a brown transparent solution. Especially, the synthetic yield of M_0.5_, M_1_, M_1.5_, and M_2_ was 93.3%, 95.5%, 95.8%, and 96.1%, respectively. Additionally, there one work reported that the synthetic yield of M_0.5_, M_1_, M_1.5_, and M_2_ was 98.1%, 98.7%, 99.2%, and 99.7%, respectively [42]. Therefore, this similarity indirectly implied the accuracy of our experiments.

#### 2.2.2. Synthesis of HAPAM and PAM

**Figure 1:**
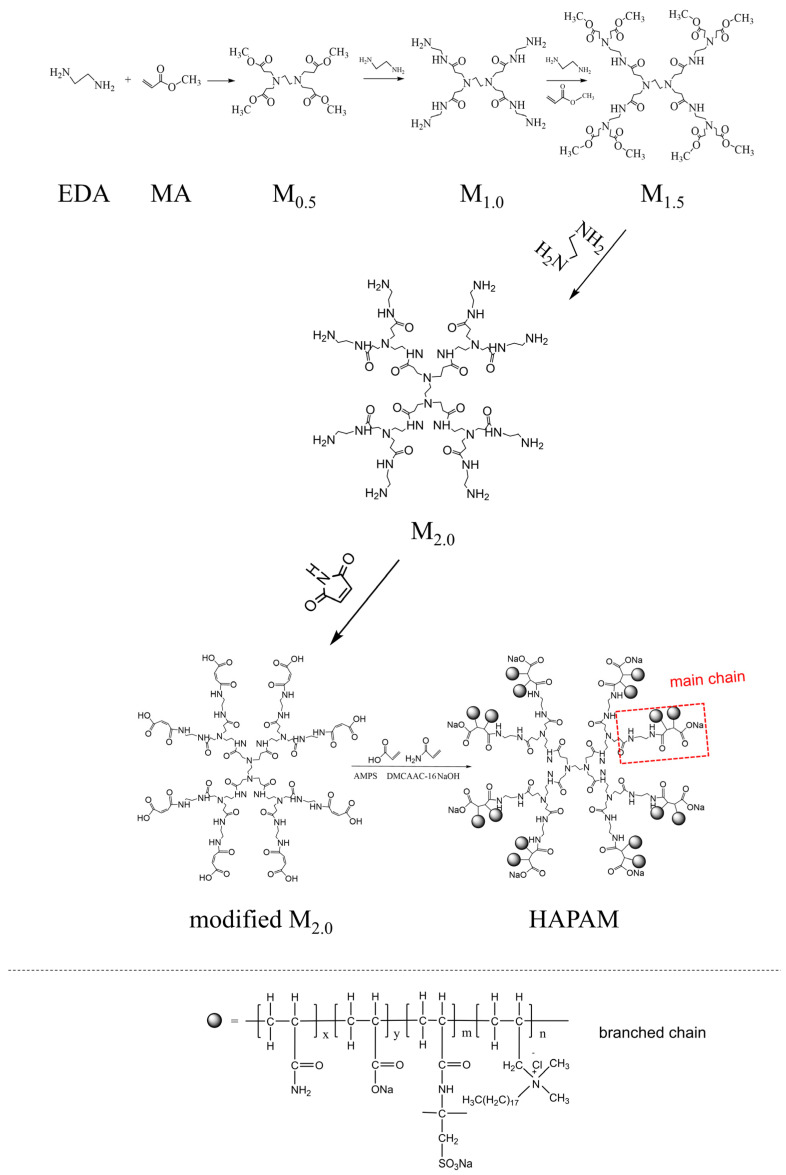
Synthetic route and structure of HAPAM.

To enable the dendritic skeleton to participate in polymerization, cis-butenedioic anhydride was used to introduce double bonds into the modified dendritic M_2.0_. Then, the detailed synthesis process of HAPAM was given as follows: a round bottom flask with three necks was added with 30 g of deionized water and 6.0 g of AA and deaerated by N_2_. NaOH was added into the solution to adjust pH = 7 after the complete dissolution of AA. Besides, the solution was added with 6.1 g of AM, 0.5 g of AMPS, 1.2 g of DMCAAC-18, and 0.5 g of M_2.0,_ respectively, stirring until complete dissolution. Again, deionized water was added to adjust the mass concentration (25 wt%) of AM, AMPS, DMCAAC-18, and M_2.0_ in the whole solution. The temperature of the solution was stabilized at 40 °C, gradually adding (NH_4_)_2_S_2_O_8_ and NaHSO_3_ (0.2 wt% and molar ratio of (NH_4_)_2_S_2_O_8_ and NaHSO_3_ was 1:1) as an initiator with stirring evenly. Finally, the above mixtures were sealed in a beaker for reaction about 4 h to obtain HAPAM. The synthesis formula of D-M-HPA is shown in Figure 1. The PAM was prepared using the same method without adding DMCAAC-18 and M_2.0_.

### 2.3. Characterization

The microstructures of HAPAM and PAM were characterized by a VEGA3SBU scanning electron microscope (SEM, Beijing Dongxiyi Technology Co., Ltd., Beijing, China). The sample preparation for SEM was given as follows: (i) the concentration of both PAM solution and HAPAM polymer solution was 1000 mg/L, (ii) 1-2 drops of the solutions were taken onto the conductive adhesive and treated in a vacuum freeze-drying oven for 24 h, (iii) after gold spraying, the samples were observed by scanning electron microscopy. The infrared spectrum of HAPAM was measured by a Fourier infrared spectrometer (FTIR-650, Tianjin Zhongke Ruijie Technology Co., Ltd., Tianjin, China). HAPAM was characterized by an FTIR-650 Fourier infrared spectrometer. The sample preparation for FTIR was given as follows: (i) Wash HAPAM with anhydrous ethanol three times, (ii) put it in the oven at 35 °C to get a solid with a crisp texture, (iii) take it out and grind it into powder, iv) evenly disperse the ground fine powder in potassium bromide and press it into a transparent sheet sample, (v) KBr should be dried before use, and the grinding particle size was less than 2 microns. The ^1^H-NMR spectrum of HAPAM was obtained by a nuclear magnetic resonance instrument (Bruker-AC-E200, Bruker, Switzerland). The sample preparation for 1H-NMR was given as follows: (i) Clean HAPAM with anhydrous ethanol 3 times to remove impurities, (ii) put it in a vacuum drying oven at 35 °C for drying, (iii) take it out and grind it into a fine powder after drying, (iv) put 1–2 mg into a nuclear magnetic tube, and (v) use 0.55 mL D_2_O to completely dissolve it.

### 2.4. Performance-Evaluation

The difference between HAPAM and PAM on thickening ability, shear-resistance, thermal endurance, and salt-resistance were evaluated via a series of static tests by measuring the apparent viscosity of HAPAM and PAM solutions using an NDJ-5S digital rotary viscometer rotor at the speed of 30 rpm (Shanghai Fangrui Instrument Co., Shanghai, China). Additionally, the interfacial tension of HAPAM and PAM solutions (with mass concentrations of 500 mg/L, 750 mg/L, and 1000 mg/L) was measured by the interfacial tension meter (Guangdong Insa Instrument Technology Co., Dongguan, China). The resistance-coefficient and residual-resistance-coefficient for 2000 mg/L of HAPAM and PAM solutions were determined using sandpack core flooding experiments at 65 °C. The sand pack core holder (Φ2.5 cm × 30 cm) was filled with quartz sand of 80–120 meshes (with a porosity of 36.69%). Before oil injection, the packed core was vacuumed with a vacuum pump to drain the core and saturated water completely. Then, the initial water permeability of the sand pack core was measured, and the porosity of the core could be calculated according to the volume of the brine absorbed by the vacuumed porous media. In addition, crude oil was injected into the sand pack core until the brine production became negligible. Secondly, the specific experimental steps of water flooding and polymer flooding were given as follows: (1) Water-driving-stage: the sand pack core holder was injected with the simulated injection water at an injection speed of 10 mL/min, recording the stabilized pressure of $P_{w1}$ (MPa). (2) Polymer-flooding-stage: 2000 mg/L of HAPAM or PAM solutions were injected into the one-dimensional sand-filling model at an injection rate of 10 mL/min, also recording the stabilized pressure of $P_{p}$ (MPa). (3) Subsequent-water-driving stage: the one-dimensional sand-filling model was injected with the simulated injection water again at an injection speed of 10 mL/min, recording the stabilized pressure of $P_{w2}$ (MPa). The resistance-coefficient (RF) and residual-resistance-coefficient (RRF) can be given as the following formula.

(1) $$RF=\frac{P_{p}}{P_{w1}}$$

(2) $$RRF=\frac{P_{w2}}{P_{w1}}$$

Moreover, the oil-displacement performance of HAPAM or PAM solutions (2000 mg/L) was also determined by the sand pack core flooding experiments. The detailed experimental steps are as follows: (1) The sand pack core holder was saturated with the simulated injection water, as given in Table 1 (water-flooding). (2) Then, it was saturated with crude oil (778.56 mPa·s) at a speed of 2 mL/min (polymer-flooding) until irreducible water saturation. After aging for 24 h, the simulated injection water was injected at a speed of 2 mL/min to displace the crude oil until the water cut reached above 95%. Again, the polymer solution was injected at a speed of 2 mL/min to obtain a water cut that reached above 95%. The EOR of the polymer solutions can be calculated by the following equation:

(3) $$EOR=E_{total}-E_{water}$$

where *EOR*, *E_total_*, and *E_water_* were *EOR* of polymer solution (%), oil recovery of water-flooding and polymer-flooding process (%), and oil recovery of water-flooding process (%).

## 3. Results and Discussion

### 3.1. Infrared Spectrum of M_0.5_, M_1.0_, M_1.5_, M_2.0_, and HAPAM

**Figure 2:**
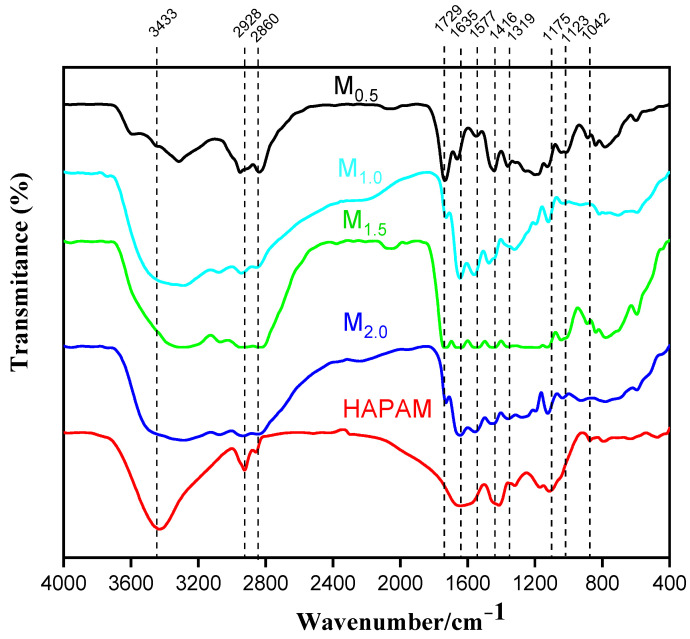
IR Spectrum of M0.5, M1.0, M1.5, M2.0, and HAPAM.

The IR spectra of M_0.5_, M_1.0_, M_1.5_, M_2.0_, and HAPAM were given in Figure 2, and the peaks were also given in Table 2, respectively. For M_1.0_, M_1.5_, and M_2.0_, the peaks near 3288~3295 cm^−1^ and 3068~3078 cm^−1^ can be ascribed to N-H stretching vibration [43,44], while the strong peak of HAPAM near 3433 cm^−1^ can be assigned to the multiple complex groups of its -CONH- and -CONH_2_. Meanwhile, the peaks of M_0.5_, M_1.0_, M_1.5_, M_2.0_, and HAPAM around 2928~2951 cm^−1^ and 2837~2860 cm^−1^ were categorized as antisymmetric and symmetric stretching vibration of C-H [45,46]. The C=O stretching vibrational peaks in the group of -COOC- for M_0.5_, M_1.0_, M_1.5_, and M_2.0_ were located at relatively stronger peaks of 1727~1731 cm^−1^ and weaker peaks of 1644~1652 cm^−1^. Meanwhile, the C=O stretching vibrations in several groups of -CONH-, -CONH_2_, and -COONa for HAPAM resulted in a strong and broad peak near 1635 cm^−1^ [47,48]. In particular, for M_0.5_, M_1.0_, M_1.5_, M_2.0_, and HAPAM, the N-H bending vibrational peaks of CONH-, the C-H in-plane bending vibrational peaks, and the C-N stretching vibrational peaks of tertiary amines were respectively appeared round 1549~1477 cm^−1^, 1416~1473 cm^−1^, and 1319~1361 cm^−1^, respectively [49,50,51]. In addition, the stretching vibrational peaks of C-C and tertiary amine C-N were observed at 1175~1199 cm^−1^ in M_0.5_, M_1.0_, M_1.5_, M_2.0_, and HAPAM [52]. Interestingly, the stretching vibrational peaks of C-O-C in M_0.5_ and M_1.5_, the stretching vibrational peaks of primary amine C-N in M_1.0_ and M_2.0_, the C-O bonds of -COONa in HAPAM, and the stretching vibrational peak of S=O in -SO_3_Na were also overlapped at this position [53,54,55]. Also, tertiary amine C-N stretching vibrational peaks of M_0.5_, M_1.0_, M_1.5_, M_2.0_, and HAPAM were observed around 1123~1127 cm^−1^ [56]. In addition, the primary amine C-N stretching vibration and N-H in-plane bending vibration stretching vibration peaks for M_1.0_ and M_2.0_, the C-O bond of -COONa in HAPAM, and the S-O stretching vibration peak in -SO_3_Na were also overlapped around this range [57,58,59]. The stretching vibration peaks of tertiary amine C-N were observed at 1036~1044 cm^−1^ for M_0.5_, M_1.0_, M_1.5_, M_2.0_ [60], and HAPAM. Meanwhile, the stretching vibration peaks of primary amine C-N and N-H in-plane bending vibration for M_1.0_ and M_2.0_ also overlapped around this position [61]. Thus, the strong characteristic peaks of -COONa and -SO_3_Na indicated the successful synthesis of HAPAM.

### 3.2. H-NMR Spectrum of HAPAM

**Figure 3:**
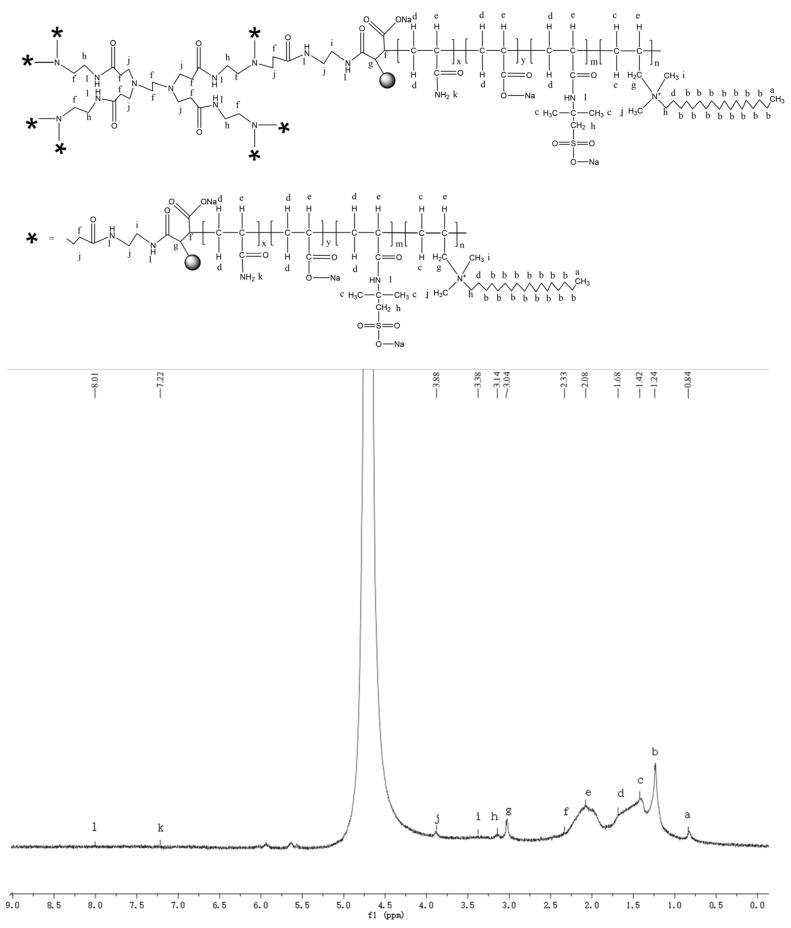
1H-NMR Spectrum of HAPAM.

The 1H-NMR spectrum of HAPAM is illustrated in Figure 3, and all the peaks have been labeled from a to l, and the positions in the HAPAM structure were indicated accordingly. **Peak a** (δ0.84) and **peak b** (δ1.24) resulted from the proton absorption peaks of -C**H**_3_ and -(C**H**_2_)- in the group of -N^+^-CH_2_-CH_2_-(C**H**_2_)_15_C**H**_3_ in the hydrophobic branched chain of DMCAAC-18 [62]. **Peak c** (δ1.42) was caused by the proton absorption peak of 
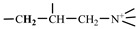
 in the hydrophobic branched chain of DMCAAC-18 and by the proton absorption peak of -C**H**_3_ in the AMPS chain. **Peak d** (δ1.68) belonged to the proton absorption peak of 
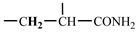
 [63], 
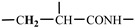
, 
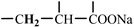
, and -CH_2_-C**H**_2_-(CH_2_)_15_CH_3_ in the chain of AA, AM, AMPS, and hydrophobic branched chain of DMCAAC-18. **Peak e** (δ2.08) was assigned to the proton absorption peak of 
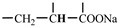
, 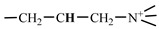, 
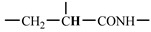
, and 
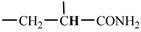
 in the branched chains. **Peak f** (δ2.33) was resulted from the proton absorption peak of 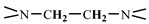 [41], 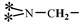, and 
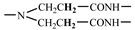
 in the branched chain-skeleton and the proton absorption peak of 
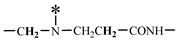
 and 
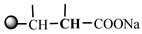
 at the branched chain. **Peak g** (δ3.04) was the proton absorption peak of 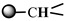 at the branched chain and the proton absorption peak of 
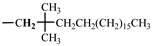
 at the hydrophobic branched chain of the DMCAAC-18 chain. The proton absorption peak of 
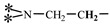
 at the branched chain skeleton, the proton absorption peak of -C**H**_2_-CH_2_-(CH_2_)_15_CH_3_ in DMCAAC-18 chain, and the proton absorption peak of -C**H_2_**SO_3_Na in the AMPS chain resulted in **Peak h** (δ3.14). The proton absorption peak of 
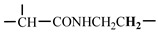
 at the branched chain and the proton absorption peak of 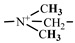 in the DMCAAC-18 chain resulted in **Peak i** (δ3.38). **Peak j** (δ3.88) belonged to the proton absorption peak of 
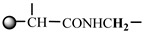
 and 
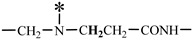
 in the branched-chain and the proton absorption peak of 
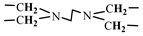
 in the branched chain-skeleton. **Peak k** (δ7.22) was the proton absorption peak of 
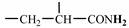
 [63] in the branched chain. **Peak l** (δ8.01) was caused by the proton absorption peak of -CO-N**H**- at the branched chain and at the branched chain skeleton. The peaks corresponded to the expected chemical structure and functional group of HAPAM, demonstrating the successful synthesis of HAPAM.

**Figure 4:**
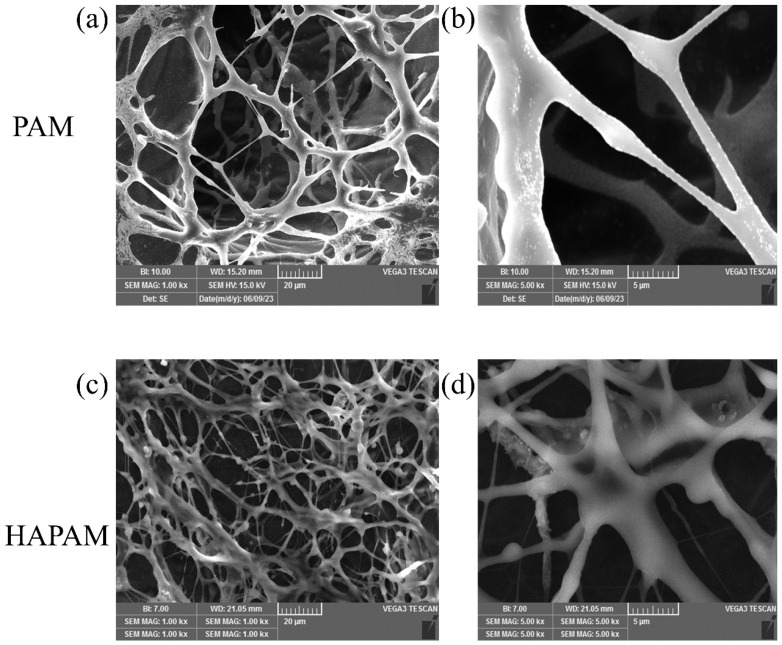
SEM observations of PAM ((**a**,**b**), magnifying ×1000 and ×5000) and HAPAM ((**c**,**d**), magnifying ×1000 and ×5000).

The microstructure of PAM solution and HAPAM solution was observed by VEGA-3SBU scanning electron microscope. The SEM observations (magnifying 1000× and 5000×) of HAPAM and PAM are shown in Figure 4. From the micro-scale structures visible in SEM, it can be found that the entanglement degree of complexes formed by the aggregation of many HAPAM polymers was significantly higher than that of PAM due to drying. In other words, compared to PAM, this phenomenon indicated that HAPAM had much denser networks, which was conducive to enhancing the rigidity and strength of molecular chains and molecular volume [64] and improving its shear resistance and sweep efficiency [65].

### 3.3. Determination of Intrinsic Viscosity and Molecular Weight

The molecular weight of a polymer can be measured from its intrinsic viscosity by a Ubbelohde viscometer at room temperature, which can be used to estimate the size of the polymer to better understand its hydrodynamic behavior. From the analysis of the structure of the synthesized hyperbranched HAPAM, it has similar branches to PAM. However, the linear and long molecular chains of HAPAM were distributed to the individual branches, reducing the fluid mechanical volume of the molecule. Thus, it can be theoretically estimated that the fluid mechanical volume of HAPAM was smaller than that of PAM. Additionally, 1 mol/L of NaCl was prepared with deionized water as the standard solution. 10 mL and 1 mol/L of NaCl solution was transferred into the Ubbelohde viscometer. The side vent tube was sealed, then the solution was gently sucked into the timing tube until the liquid reached about 5 mm above the upper timing mark. Then, loosen the side vent tube to allow the liquid to fall freely and measure the flow time. To measure the flow time, start the stopwatch when the meniscus drops below this upper timing mark, measuring the time for the meniscus to pass from the upper timing to the bottom timing mark (stop the stopwatch when the meniscus drops below the bottom timing mark). The average value of the three tests was taken as the flow time of the standard solution (10 mL and 1 mol/L of NaCl solution). Similarly, 10 mL of 500 mg/L polymer solutions (prepared with 1 mol/L of NaCl solution) was used to determine the flow time, as shown in Table 3. For a given capillary viscometry, it provides only a relative measure of the viscosity and not an absolute one, as shown in Equation (4). The intrinsic viscosity of the polymer can be obtained by extrapolating the plot of ln*η*_r_/*C* vs. *C* to C = 0, as given in Equation (5). According to the general form of the Mark-Houwink relation, the Viscosity-average molecular weight of the polymer can be obtained in units g/mol by Equation (5).

(4) $$\eta_{r}=\frac{\eta }{\eta_{0}}=\frac{t}{t_{0}}$$

(5) $$\left[\eta \right]=\underset{C\to 0}{\lim}\left(\frac{1}{C}\frac{\eta }{\eta_{0}}\right)=KM^{\alpha }$$

$\eta_{r}$: relative viscosity, dimensionless; η (cm^2^ s^−1^) and *t* (s): viscosity and flow time of the polymer solution; $\eta_{0}$ (cm^2^ s^−1^) and $t_{0}$ (s): viscosity and flow time of NaCl standard solution; [*η*]: intrinsic viscosity, (cm^3^/g); *C*: concentration of polymer solution, (g/cm^3^); *K*: a constant of the Ubbelohde viscometer, (cm^3^ mol^−1^); *M*: the Viscosity-average molecular weight of polymer (g/mol); α: a dimensionless constant, (0.5 < a < 0.8).

**Table 3:** Flow time measured by Ubbelohde viscometer.

Mass Concentrations	1st (s)	2nd (s)	3rd (s)	*t*_average_ (s)
1 mol/L of NaCl	112.9	112.6	112.7	112.7
500 mg/L of PAM + 1 mol/L of NaCl	167.2	167.3	167.5	167.3
500 mg/L of HAPAM + 1 mol/L of NaCl	143.5	143.8	143.6	143.6

According to experimental data and calculation, the intrinsic viscosity of PAM and HAPAM was 844 cm^3^/g and 500 cm^3^/g, respectively. Meanwhile, the Viscosity-average molecular weight of PAM and HAPAM was 3.96 × 10^6^ g/mol and 1.79 × 10^6^ g/mol, respectively. It is clear that the intrinsic viscosity and molecular weight of HAPAM were smaller than those of PAM. Due to the dendritic structure, the molecular chains of HAPAM were distributed over more branches, resulting in a smaller hydrodynamic volume per unit of polymer molecule in solution smaller than that of PAM. The smaller hydrodynamic volume possibly contributed to HAPAM having a stronger shear resistance at high speeds through small pores.

### 3.4. Thickening Capacity of HAPAM

**Figure 5:**
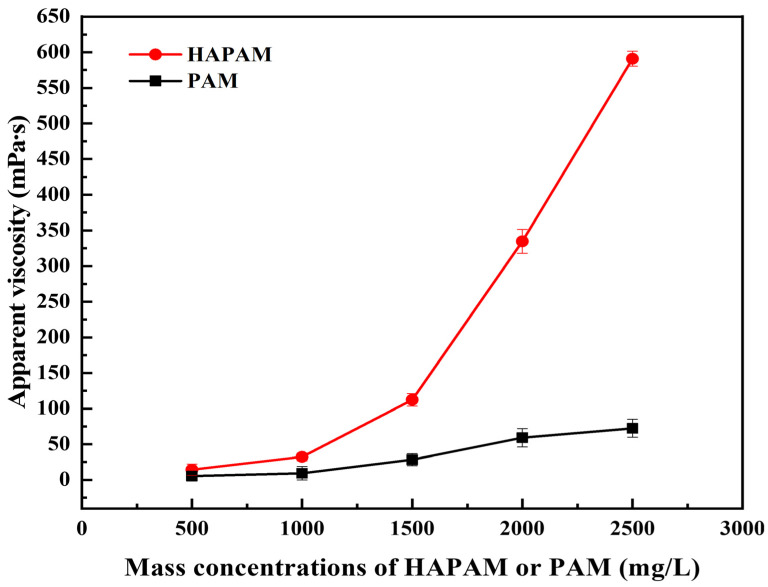
Thickening ability of HAPAM and PAM.

The comparative thickening performance of HAPAM and PAM solutions with different concentrations is shown in Figure 5. It can be found that the overall trend of the apparent viscosity for both HAPAM and PAM solutions was increased with increasing mass concentrations. A noticeable thickening capacity behavior was observed for HAPAM. Specifically, after the mass concentration reached 1500 mg/L, the apparent viscosity of HAPAM was significantly increased with the mass concentration, and the apparent viscosity of HAPAM solution reached 590.86 mPa·s at the 2500 mg/L, which was about six times higher than that of PAM. The excellent thickening ability of HAPAM was caused by the fact that HAPAM has a spatial three-dimensional network structure and hydrophobic groups, which can be confirmed by SEM observations (Figure 4), 1H-NMR (Figure 3), and IR spectrum (Figure 2). When the concentration of hydrophobic groups exceeded a specific concentration (critical association concentration) [66,67], the association of the main chains for HAPAM was gradually converted from intramolecular association to intermolecular association, resulting in a significant increase in the thickening capacity of HAPAM solution.

### 3.5. Shear-Resistance of HAPAM

**Figure 6:**
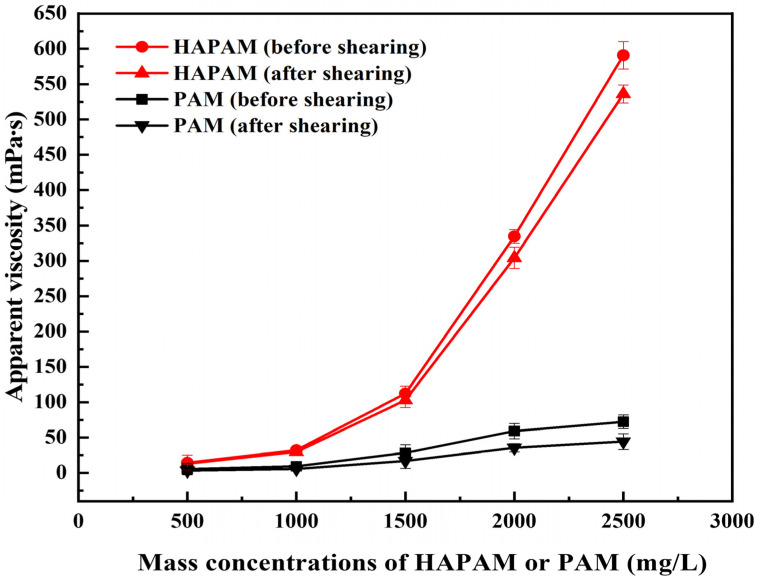
Shear-resistance of HAPAM and PAM.

**Figure 7:**
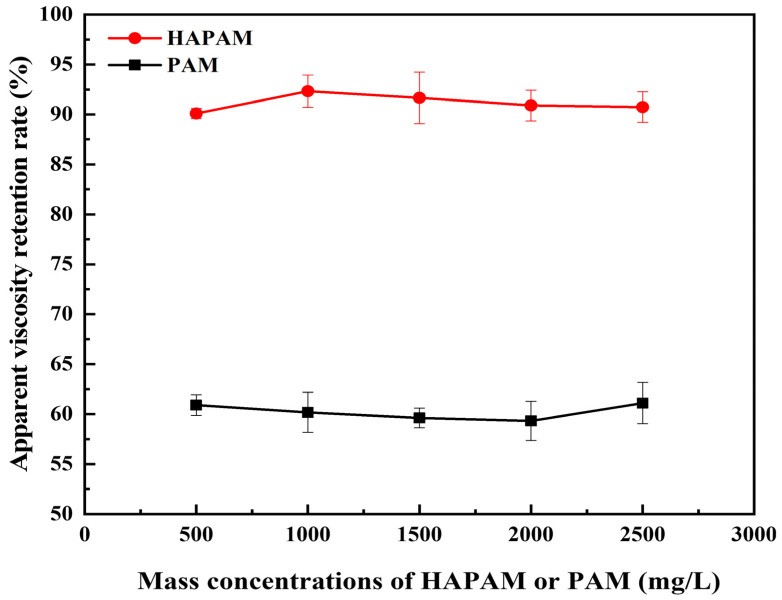
Viscosity-retention of HAPAM and PAM.

HAPAM and PAM solutions prepared with mass concentrations of 500 mg/L, 1000 mg/L, 1500 mg/L, 2000 mg/L, and 2500 mg/L were sheared for 30 s using an XF crusher (Guangdong Xinfei Fashion Co., Shenzhen, China) at 20,000 r/min. After removing bubbles, the apparent viscosity of HAPAM and PAM solutions was measured before and after shearing at 65 °C. The shear-resistance and viscosity-retention of HAPAM and PAM solutions are shown in Figure 6 and Figure 7, respectively. After 30 s shearing under the same experimental condition, the viscosity retention rate of PAM was about 60%, while HAPAM had a higher shear resistance with a viscosity retention rate of over 90%. The higher shear resistance of HAPAM may result from the highly entangled spatial three-dimensional network structure of HAPAM (as shown in Figure 4), which was greatly conducive to viscosity recovery and high viscosity retention rate.

### 3.6. Temperature-Resistance of HAPAM

**Figure 8:**
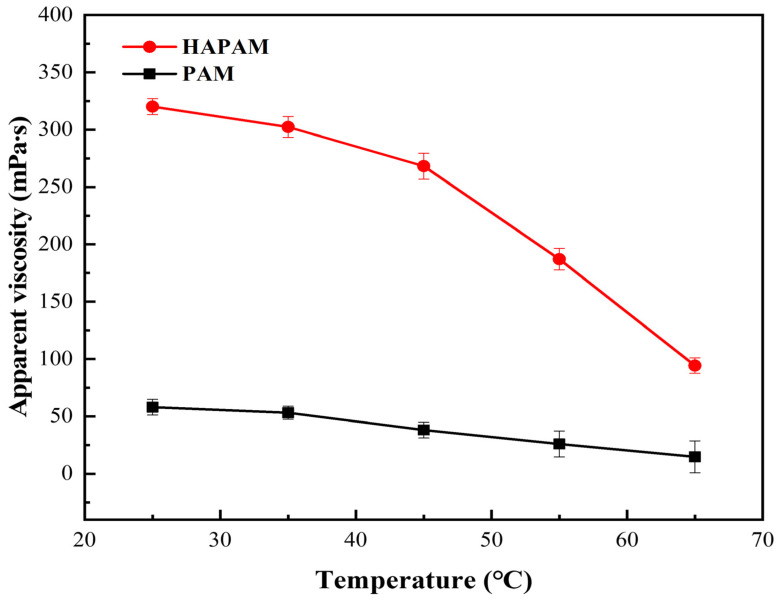
Temperature-resistance of HAPAM and PAM.

2000 mg/L of HAPAM and PAM solutions were stabilized at different temperatures for 24 h, and their apparent viscosity was measured, as shown in Figure 8. Clearly, it can be obtained that the apparent viscosity of both HAPAM and PAM solutions decreased with the increase of the temperature. Significantly, the apparent viscosity of both HAPAM and PAM solutions decreased slowly before 35 °C but rapidly decreased after 35 °C. Especially the apparent viscosity of the HAPAM solution was about 100 mPa·s at 65 °C, which was about 10 times higher than that of the PAM solution (12 mPa·s). This may be illustrated by the fact that the winding degree of HAPAM molecular chains was weakened by the enhancement of temperature. Since the mutual entanglement between macromolecular chains was caused by the Browne motion of molecules. With the increase in temperature, the thermal motion of hydrophobic chains and water molecules was intensified, which damaged the entanglement between molecular chains to a certain extent, which was not conducive to hydrophobic association and thus reduced the viscosity. At low temperatures, the destructive effect of thermal motion was not obvious. After reaching a certain temperature, the damage of thermal motion rapidly weakened the winding degree of molecular chains. Thus, the viscosity decreased significantly. Comparatively, HAPAM displayed better temperature-resistance. The viscosity retention rate of HAPAM (59.1%) at 65 °C was higher than that of PAM (48.9%), which can enable us to demonstrate the temperature-resistance of HAPAM was better than that of PAM [68].

### 3.7. Salt-Resistance of HAPAM

**Figure 9:**
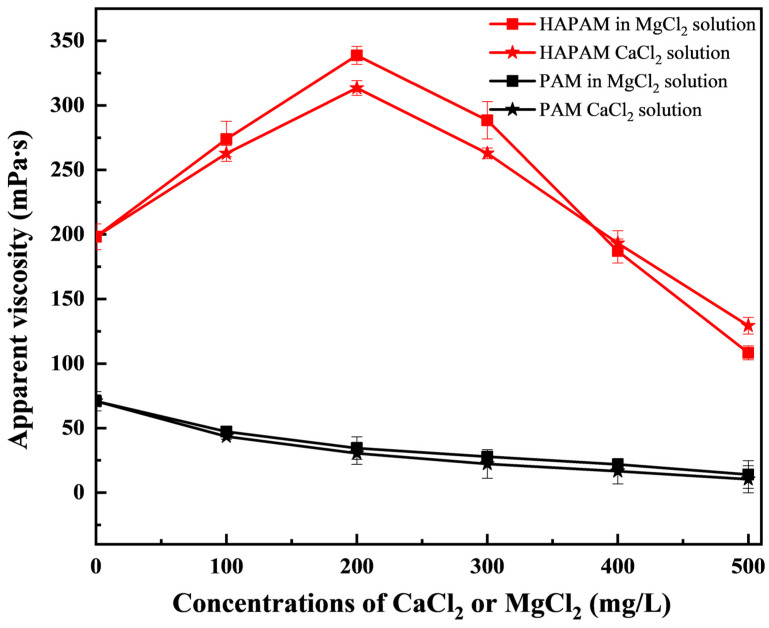
The salt-resistance of HAPAM and PAM in CaCl_2_ and MgCl_2_ solutions.

2000 mg/L of HAPAM and PAM solutions were added with different kinds of salts (NaCl, CaCl_2_, and MgCl_2_) to investigate the salt resistance. Then, HAPAM and PAM solutions were stabilized at 65 °C for 30 min, and their apparent viscosity was measured. The comparative performance of salt resistance for HAPAM and PAM solutions is shown in Figure 9, respectively. Clearly, the apparent viscosity of PAM was rapidly decreased with the increase of salinity, and then it was kept at a low value. PAM was sensitive to salt, which was attributed to the fact that the presence of cations in the salt-containing solution played a shielding effect on the electricity of carboxylic acid groups generated from the hydrolysis of amide groups, weakening the electrostatic repulsion, resulting in the change of molecular chain from an extended state to a curled state. Therefore, the decrease of the hydrodynamic volume occupied in the space and the corresponding decrease in apparent viscosity of the PAM solution was observed [69]. However, at low salinity of HAPAM solution (C_CaCl2_ ≤ 200 mg/L and C_MgCl2_ ≤ 200 mg/L), the apparent viscosity of HAPAM solution gradually increased with the increase of the salinity, obtaining the maximum of 313.49 mPa·s (in CaCl_2_ solution) and 338.69 mPa·s (in MaCl_2_ solution), respectively. In the HAPAM solution, when added with salt, the association between molecular groups and molecular chains was shielded by ions, resulting in the enhancement of the interaction between solvent molecules. Hence, the molecular chains of HAPAM became stretched, which was favorable to the formation of hydrophobic micropores [70]. Therefore, the apparent viscosity did not decrease with the increase in the concentration of added salt but rather increased with the increase in the concentration of added salt. Meanwhile, at the high salinity of HAPAM solution (C_CaCl2_ > 200 mg/L and C_MgCl2_ > 200 mg/L), the apparent viscosity of HAPAM solution slowly decreased with the increase of the salinity degree. Clearly, compared with PAM, HAPAM exhibited better salt-tolerance-ability and long-term stability.

### 3.8. Interfacial Tension of HAPAM

HAPAM and PAM solutions (with mass concentrations of 500 mg/L, 750 mg/L, and 1000 mg/L) were prepared for measuring interfacial tension. The interfacial tensions were measured at the interface between crude oil and PAM or HAPAM solutions at 25 °C. As shown in Table 4, the interfacial tension for PAM or HAPAM solutions gradually decreased with the increase of its mass concentration. The decreased trend of interfacial tension of the oil|HAPAM interface was mainly ascribed to the directional distribution of the hydrophilic and lipophilic groups in the oil-polymer interface layer. As the n concentration of HAPAM increased, the number of hydrophilic and lipophilic groups arranged on the interface layer increased, resulting in the greater ability to reduce the interfacial tension. Significantly, HAPAM exhibited a stronger ability to reduce the interfacial tension of the oil|HAPAM interface than that of PAM solution under the same experimental condition. Moreover, the amphiphilic property of HAPAM not only enables crude oil to be stripped from the surface of the porous medium but also effectively avoids chromatographic separation with excellent injectability and flow-controlling ability [71].

### 3.9. Resistance-Coefficient and Residual-Resistance-Coefficient of HAPAM

The resistance-coefficient (RF) and residual-resistance-coefficient (RFF) of PAM and HAPAM solutions (2000 mg/L) are shown in Table 4, respectively. RF and RFF were two important technical indicators for evaluating and understanding the oil-displacement mechanism of the polymers [72,73], which were also important indicators for indoor screening and evaluation of polymers. RF was used to describe the increase in water viscosity and retention due to the presence of polymer in the porous media. Moreover, for a low RF value, it implied that the polymer can be easily injected in the field without pressure increase concerns. Additionally, RFF was an indicator of the ability of polymer solution to reduce the permeability of pore media. The higher the RFF value, the more significant the adsorption and retention phenomenon of the polymer in porous media, which indicated the more helpful for improving the oil-displacing ability of the polymer. As given in Table 5, it can be obtained that the RF and RFF of PAM solutions (2000 mg/L) were 11.22 and 1.45, respectively. Similarly, the RF and RFF of HAPAM solutions were 16.77 and 2.80, respectively. Obviously, the RF and RFF of the HAPAM solution were higher than those of the PAM solution, so HAPAM should be superior to PAM in improving swept volume and building percolating resistance. The strong percolating resistance to the porous medium was readily facilitated by the high resistance coefficient of the HAPAM solution, allowing them to spread into more pores. Also, the permeability of the porous medium was greatly affected by the high residual resistance coefficient of the HAPAM solution, which can expand the coverage range of subsequent water flooding.

### 3.10. EOR of HAPAM Solutions

**Figure 10:**
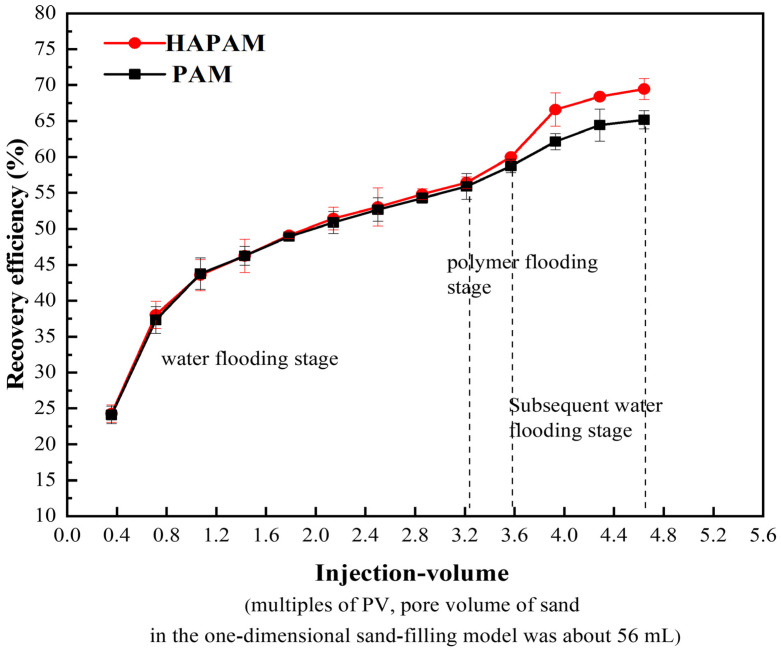
Variation of crude oil recovery rate with injection-volume during displacement of HAPAM and PAM (2000 mg/L).

The oil-displacing performance of PAM and HAPAM solutions (2000 mg/L) is given in Figure 10. As the permeability of the sand pack core holder was approximately 0.293 μm^2^, following water cut to 95% for water flooding, the recovery efficiency increased with the increase of injection volume (PV) of the injected water. The recovery efficiency was about 56.43% at 3.2PV. During 3.2–3.6PV, using HAPAM solution at the same injection rate, the recovery efficiency was 60.00%, with an increase of 3.57%. The subsequent water flooding could further increase the oil recovery, reaching 69.46%, with an increase of 3.57%. Overall, the enhanced oil recovery (EOR) of PAM and HAPAM solutions was 9.29% and 13.03%, respectively. Obviously, HAPAM exhibited a stronger oil recovery ability than that of PAM. The higher EOR of HAPAM solution can be assigned to the improved sweep efficiency and sweep volume by the excellent thickening performance, shear-resistance, and hydrophobic association of HAPAM solution. In addition, HAPAM had surface activity to reduce the interfacial tension at the interface between the crude oil and HAPAM solution, which was also beneficial for improving oil washing efficiency.

## 4. Conclusions

In this study, a novel hyperbranched polymer with polyacrylamide side chains, HAPAM, was successfully synthesized by polymerization of AA, AM, AMPS, DMAAC-18, and M_2.0_ in aqueous solution at 40 °C and pH = 7 for 4 h, using (NH_4_)_2_S_2_O_8_-NaHSO_3_ as initiator. The properties of HAPAM solution were superior to those of PAM solution, regarding thickening ability, shear-resistance, temperature-resistance, salt-resistance, the ability to reduce the interfacial tension between polymer solution and crude oil, and oil-displacement-efficiency. The excellent performance of HAPAM demonstrated the effectiveness and possibility of introducing hydrophobic groups (dimethyl octadecyl ammonium chloride, DMCAAC-18) and hydrophilic groups (AA, AM, and AMPS, abbreviated from 2-acrylamido-2-methyl-1-propanesulfonic acid) into the polymer chain to simultaneously enhance the shear-resistance and avoid the phenomenon of chromatographic separation. In addition, the successful synthesis of HAPAM and this novel synthetic scheme are important inspirations for the development of new high-performance oil-displacing agents.

## Figures and Tables

**Table 1:** Compositions of ions in the simulated injection water.

Ions	Na^+^	K^+^	Ca^2+^	Mg^2+^	HCO_3_^−^	SO_4_^2−^	Cl^−^
Concentrations (mg/L)	667	28	20	13	379	68	870

**Table 2:** Main peaks obtained from FTIR of M0.5, M1.0, M1.5, M2.0, and HAPAM.

No.	1	2	3	4	5	6	7	8	9	10	11	12	13	14
Peaks (wavelength/cm^−1^) of M 0.5		3315			2951	2837	1734	1662		1442	1361	1199	1126	1043
Peaks (wavelength/cm^−1^) of M 1.0			3290	3078	2943	2845	1727	1644	1561	1473	1321	1194	1120	1039
Peaks (wavelength/cm^−1^) of M 1.5			3295	3068	2946	2844	1731	1652	1549	1446	1360	1194	1127	1044
Peaks (wavelength/cm^−1^) of M 2.0			3288	3074	2943	2847	1729	1644	1556	1459	1359	1196	1123	1036
Peaks (wavelength/cm^−1^) of HAPAM	3433				2928	2860		1635	1577	1416	1319	1175	1123	1042

**Table 4:** Interfacial tension at the interface between HAPAM/PAM and crude oil.

Mass Concentrations	PAM	HAPAM
	Interfacial Tension (mN/m) ± Standard Deviations	
500 mg/L	22.94 ± 0.05	19.95 ± 0.02
750 mg/L	21.45 ± 0.05	18.64 ± 0.03
1000 mg/L	19.87 ± 0.03	15.84 ± 0.03

**Table 5:** Experimental parameters for measuring RF and RFF of HAPAM and PAM.

	Pressure in Water-Driving-Stage (MPa)	Pressure in Polymer-Flooding-Stage (MPa)	Pressure in Subsequent-Water-Driving-Stage (MPa)	RF	RFF
2000 mg/L of PAM	0.0094	0.1057	0.0137	11.22	1.45
2000 mg/L of HAPAM	0.0122	0.2049	0.0342	16.77	2.80

## Data Availability

Data are contained within the article.
